# The correlation between serum total bile acid and alanine aminotransferase of pregnant women and the disorders of neonatal hyperbilirubinemia-related amino acid metabolism

**DOI:** 10.1186/s12884-023-06226-9

**Published:** 2024-01-03

**Authors:** Xizhenzi Fan, Huijuan Rong, Yingying Wang, Mingwei Li, Wenhui Song, Achou Su, Tianxiao Yu

**Affiliations:** 1 Research center for clinical medical sciences, Hebei key laboratory of maternal and fetal medicine, The Fourth Hospital of Shijiazhuang, Shijiazhuang, 050000 China; 2 Department of Nursing, The Fourth Hospital of Shijiazhuang, Shijiazhuang, 050000 China; 3 Department of Functional Region of Diagnosis, The Fourth Hospital of Hebei Medical University, Shijiazhuang, 050000 China

**Keywords:** Alanine aminotransferase, Amino acid metabolism, Total bile acid, Neonatal hyperbilirubinemia

## Abstract

**Background:**

To explore the association between liver metabolism-related indicators in maternal serum and neonatal hyperbilirubinemia (NHB), and further investigate the predictive value of these indicators in NHB-related amino acid metabolism disorders.

**Methods:**

51 NHB and 182 No-NHB newborns and their mothers who treated in the Fourth Hospital of Shijiazhuang from 2018 to 2022 were participated in the study. The differences in clinical data were compared by the Mann-Whitney U test and Chi-square test. Multivariate logistic regression was used to analyze the relationship between maternal serum indicators and the occurrence of NHB. The correlation analysis and risk factor assessment of maternal serum indicators with NHB-related amino acid metabolic disorders were performed using Spearman correlation analysis and multivariate logistic regression.

**Results:**

Compared to the non NHB group, the NHB group had higher maternal serum levels of alanine aminotransferase (ALT), aspartate aminotransferase (AST), ALT/AST, and total bile acid (TBA), while lower levels of serum albumin (ALB), total cholesterol (TC) and high-density lipoprotein (HDL). The levels of alanine (ALA), valine (VAL), ornithine (ORN), and proline (PRO) in the newborns were reduced in NHB group, while arginine (ARG) showed a tendency to be elevated. Multiple logistic regression analysis showed that maternal ALT, AST, ALT/AST, and TBA levels were all at higher risk with the development of NHB, whereas ALB, TC, and HDL levels were negatively associated with NHB development. Increasing maternal TBA level was associated with lower ALA (r=-0.167, *p* = 0.011), VAL (r=-0.214, *p* = 0.001), ORN (r=-0.196, *p* = 0.003), and PRO in the newborns (r=-0.131, *p* = 0.045). Maternal ALT level was negatively associated with ALA (r=-0.135, *p* = 0.039), VAL (r=-0.177, *p* = 0.007), ORN (r=-0.257, *p* < 0.001), while ALT/AST was positively correlated with ARG (r = 0.133, *p* = 0.013). After adjustment for confounding factors, maternal serum TBA and ALT were the independent risk factor for neonatal ORN metabolic disorders [(adjusted odds ratio (AOR) = 0.379, 95%CI = 0.188–0.762, *p* = 0.006), (AOR = 0.441, 95%CI = 0.211–0.922, *p* = 0.030)]. Maternal ALT level was an independent risk factor for neonatal VAL metabolic disorders (AOR = 0.454, 95%CI = 0.218–0.949, *p* = 0.036).

**Conclusions:**

The levels of high TBA, ALT, AST, and low HDL, TC of maternal were associated with the risk of NHB. Maternal TBA and ALT levels were independent risk factors for NHB-related amino acid disturbances which have value as predictive makers.

**Supplementary Information:**

The online version contains supplementary material available at 10.1186/s12884-023-06226-9.

## Introduction

Neonatal hyperbilirubinemia (NHB) is the most common neonatal disease, with a prevalence rate of 20-40%, which is increasing year by year [1]. NHB threatens fetal health and increases the risk of long-term disease. As the liver function of the newborn is not yet fully developed, and the compensatory increase in the number of red blood cells after birth can easily lead to the accumulation of inadequate bilirubin metabolism in the body [2, 3]. Many studies report that newborns with NHB have not only experience neurological damage [4, 5], but also cardiovascular [6], gastrointestinal [7], and urinary damage [8, 9], which poses a serious threat to neonatal growth and health.

It is generally accepted that apart from blood incompatibilities, breastfeeding, prematurity and family history of NHB [10], liver dysfunction such as intrahepatic cholestasis of pregnancy (ICP) in mothers during pregnancy leading to disturbances of alanine aminotransferase (ALT), aspartate aminotransferase (AST), and total bile acid (TBA) markers are also risk factors for the development of NHB [11]. Juusela et al. revealed a statistically significant correlation between TBA and AST levels and perinatal outcomes. They confirmed AST levels strongly predicted hyperbilirubinemia in the neonates with receiver operating characteristic (ROC) models [12]. Although the relationship between maternal liver dysfunction during pregnancy and the risk of NHB has attracted the attention of many scholars, it has not been specified yet whether maternal liver dysfunction is responsible for NHB due to the lack of clarity on the pathogenesis of NHB and the poor integrity of clinical data.

Amino acid is an important nutrient element for the growth and development of newborns [13, 14]. As essential amino acids, alanine (ALA) and valine (VAL) not only maintain normal physiological activities in the body, but also participate in the liver repair process [15, 16], ornithine (ORN), proline (PRO), and arginine (ARG) can improve liver function, enhance immunity and maintain positive nitrogen balance, respectively [17–19]. Therefore, disorders of amino acid metabolism will seriously affect the normal physiological development of newborns. In recent years, with the study of NHB-related adverse outcomes, NHB-related amino acid metabolism disorders have also attracted widespread interest. However, the current assessment of amino acid metabolism disorders in newborns is primarily conducted by testing amino acid levels in the heel blood of newborns at 3–4 days [20], which greatly limits the opportunity for early clinical intervention during fetal and neonatal periods. As the main site of amino acid metabolism, impaired liver function might be an early marker of abnormal amino acid metabolism in the body. Fetal and pregnant women exchange and transfer substances through the umbilical cord. Therefore, whether the abnormal liver function of pregnant women is related to NHB-related amino acid metabolism disorders, and whether fetal or newborn amino acid metabolism levels can be predicted by detecting serum liver function markers of pregnant women to achieve the goal of early detection and intervention is what we want to explore in this study.

## Materials and methods

### Study participants and data collection

The population for this retrospective study was composed of pregnant women and their newborns who came to The Fourth Hospital of Shijiazhuang, China for their obstetric examination from January 2018 to January 2022. All participants signed a written informed consent form before entering the study. The study was approved by the Ethics Review Committee of The Fourth Hospital of Shijiazhuang (No. 20,230,034). The neonatal gender, birth weight, and biochemical indicators are obtained by consulting the medical records system. The basic characteristics of their pregnant women, including age, pre-pregnancy body mass index (BMI), number of births, gestational weight gain gestational week, and the results of pregnancy tests were obtained through questionnaires and electronic medical records. The Eligible participants were newborns who met NHB diagnostic criteria [21] and their mothers had a singleton natural pregnancy, without previous and current medical history of chronic diseases such as type 1 or type 2 diabetes, hypertension, malignancy, hypothyroidism, acute or chronic inflammatory or infectious diseases, fever, iron deficiency anemia. Finally, 51 newborns with NHB and had complete data were included, and 182 newborns with No-NHB were enrolled as the control group in the same period.

The whole blood samples for detecting amino acids are collected from the heel of newborns who have been fully fed for more than 72 h after birth. All blood samples from the mothers were fasted for more than 8 h, collected under sterile conditions, and then tested by a laboratory physician within 2 h. The liver function and blood lipid levels were measured using a fully automated biochemical analyzer (Cobas 701, Roche), and the amino acid content in the whole blood of neonates was detected by high performance liquid mass spectrometry-tandem mass spectrometry (HPLS-MS, ABI4500).

### Diagnosis of NHB

According to guidelines published by the American Academy of Pediatrics (AAP) in 2004, hyperbilirubinemia was diagnosed when percutaneous bilirubin exceeded the 95th percentile of the Bhurani column chart monitoring time point during bilirubin monitoring or phototherapy of the neonate [21].

### Statistical analysis

SPSS 22.0 was used to perform a statistical analysis of the data. Median (range) and frequencies (percentage) were described as the continuous variables and categorical variables respectively. The Mann-Whitney U test was used to compare the significant difference between non-normal continuous variables, and the categorical variables were compared by Chi-square test. Multivariate logistic regression was used to evaluate the relationship between NHB risk and ALT, AST, ALT/AST, TBA, albumin (ALB), total cholesterol (TC), and high-density lipoprotein (HDL), respectively. The association between the levels of the above indicators and amino acid levels in neonatal with NHB was evaluated by Spearman correlation analysis. Then, we performed multivariate logistic regression to confirm the independent risk factors for NHB-related amino acid metabolism disorders. All statistical analyses with significant differences were considered to be *p* < 0.05.

## Result

According to the occurrence of neonatal hyperbilirubinemia, we divided the participants into two groups of no-neonatal hyperbilirubinemia (No-NHB) group and neonatal hyperbilirubinemia (NHB) group. Table 1 showed the general and pregnancy outcomes of newborns and their mothers. We found that newborns with hyperbilirubinemia had significantly lower body weights than the No-NHB group, and the incidence of premature delivery and hydramnios were significantly higher than that of the control group. Mann-Whitney U test and the Chi-square test of maternal demographic information and pregnancy outcomes for newborns with NHB showed that these pregnant women were older, more likely to have a lower gestational weight gain, gestational week, and had a higher risk of cesarean section and preeclampsia.

**Table 1:** General and clinical information of participants

	Variables	No-NHB	NHB	*p*-value
Neonatal	n	182	51	
	Male neonates (n%)	104 (57.1)	26 (51.0)	0.434 ^b^
	Female neonates (n%)	78 (42.9)	25 (49.0)	
	Neonatal birth weight (grams)	3200(2850–3500)	2350(1650–3522)	< 0.001 ^a^
	Preterm (n%)	15(8.2)	30(58.8)	< 0.001 ^b^
	Hydramnios (n%)	15(8.2)	11(21.6)	0.008 ^b^
Maternal	Age (years)	29.0 (27.0–32.0)	31.0 (28.0–33.0)	0.021 ^a^
	Pre pregnancy BMI (kg/m^2^)	21.0 (19.4–23.2)	21.0(19.7–23.7)	0.984 ^a^
	Gestational weight gain (kg)	14.8 (12.0–18.0)	12.0 (10.0–17.0)	0.046 ^a^
	Gestational week (week)	39.3(38.3–40.0)	36.1 (34.0–39.0)	< 0.001 ^a^
	Nulliparous (n%)	123 (67.6)	27 (52.9)	0.054 ^b^
	Cesarean section (n%)	87 (47.8)	35 (68.6)	0.008 ^b^
	Preeclampsia (n%)	21(11.5)	14(27.5)	0.005 ^b^

BMI, body Mass Index; NHB, neonatal hyperbilirubinemia

All continuous variables in the table were given as the medians (quartile1-quartile3), the categorical variables were presented as frequency (%)

The differences between the No-NHB and NHB groups were obtained using the Mann-Whitney U test (a) and the Chi-square test (b), *p* < 0.05 was considered to be significant

Next, we tested the liver enzymes and lipid metabolism indices of pregnant women in both groups (Table 2) and found that maternal with NHB newborns tend to have higher ALT, AST, and ALT/AST levels and lower levels of ALB than No-NHB group (*p* < 0.05). It is noteworthy that the maternal TBA level in the NHB group was approximately 6-fold higher than those in the control group (*p* < 0.05). In addition, we also found that the levels of TC and HDL were significantly lower in pregnant women with NHB newborns (*p* < 0.05). NHB can induce neonatal amino acid disorder which was detected by comparing the amino acid content in heel blood collected from 72 h after birth in our study. We found that the levels of ALA, VAL, ORN, and PRO in neonatal blood were significantly reduced in the NHB group, and ARG showed a tendency to be elevated (*p* < 0.05), while no significant differences were found in other amino acids.

**Table 2:** Comparison of neonatal amino acids and maternal laboratory biochemical indices of participants

	Variables	No-NHB	NHB	*p*-value
Neonatal	ALA (µmol/L)	265.6 (227.6-316.1)	251.8(189.1-287.3)	0.031
	VAL (µmol/L)	137.9 (117.4-152.6)	118.5 (106.6-139.1)	0.001
	ORN (µmol/L)	94.5 (75.7-115.6)	78.6 (60.1-101.1)	0.003
	ARG (µmol/L)	7.7 (3.8–12.6)	11.2 (6.1–15.7)	0.009
	LEU + ILE (µmol/L)	146.3 (125.6-169.7)	139.6 (120.9-160.6)	0.133
	MET (µmol/L)	24.0 (19.4–28.8)	23.9 (19.7–30.5)	0.732
	PHE (µmol/L)	52.4 (44.3–58.9)	49.5 (41.5–55.7)	0.081
	TYR (µmol/L)	93.4 (68.5–113.0)	83.2 (50.4-116.4)	0.096
	CIT (µmol/L)	12.7 (10.5–14.9)	13.8 (11.2–17.6)	0.078
	PRO (µmol/L)	438.5 (358.7–532.0)	370.8 (325.0-443.9)	0.001
Maternal	ALT (U/L)	10.0 (7.0–13.0)	14.5 (9.0-62.3)	< 0.001
	AST(U/L)	17.0 (15.0-22.8)	19.5 (16.0–51.0)	0.010
	ALT/AST	0.6 (0.5–0.7)	0.8 (0.5–1.2)	< 0.001
	TBIL (µmol/L)	7.9 (6.4–10.6)	8.6 (6.4–11.0)	0.676
	DBIL (µmol/L	1.3 (1.0-1.9)	1.7 (1.0-2.2)	0.607
	IBIL (µmol/L)	6.5 (5.4–8.4)	6.7 (5.2–9.4)	0.685
	TBA (µmol/L)	3.0 (1.6–13.6)	18.0 (3.2–36.0)	< 0.001
	TP (g/L)	61.9 (59.2–66.0)	61.6 (57.5–64.5)	0.057
	ALB (g/L)	36.4 (35.1–37.8)	35.0 (31.3–37.1)	< 0.001
	TG (mmol/L)	3.1 (2.5–3.8)	2.9 (2.0-4.5)	0.159
	TC (mmol/L)	6.3 (5.5-7.0)	5.7 (4.8–6.9)	0.025
	HDL (mmol/L)	1.9 (1.7–2.2)	1.7 (1.3–2.1)	0.004
	LDL (mmol/L)	3.7 (3.0-4.2)	3.2 (2.7-4.0)	0.099

ALA, alanine; VAL, valine; ORG, ornithine; ARG, arginine; LEU + ILE, leucine + isoleucine; MET, methionine; PHE, phenylalanine; TYR, tyrosine; CIT, citrulline; PRO, proline; ALT, alanine aminotransferase; AST, aspartate aminotransferase; TBIL, total bilirubin; DBIL, direct bilirubin; IBIL, indirect bilirubin; TBA, total bile acid; TP, serum total protein; ALB, serum albumin; TG, triglyceride; TC, total cholesterol; HDL, high-density lipoprotein; LDL, low-density lipoprotein

All continuous variables in the table were given as the medians (quartile1-quartile3) and we used Mann-Whitney U test to compare the differences between the No-NHB and NHB groups, *p* < 0.05 was considered to be significant

Abnormal liver function in pregnant women, such as ICP, can trigger adverse pregnancy outcomes in the newborns, including hyperbilirubinemia [12]. Similar to these reports, our study found that pregnant women of NHB newborns indeed show significant liver dysfunction such as high levels of ALT, AST, ALT/AST and TBA, and low ALB level (Table 2). Therefore, we applied multivariate logistic regression analyses to further identify the maternal ALT, AST, ALT/AST, and TBA levels were associated with higher risk for the development of NHB, whereas ALB, TC, and HDL levels were negatively correlated with the development of NHB (Sup 1).

In Table 2, we found that the amino acid metabolism of newborns in the NHB group was disturbed, and further confirmed that indices such as TBA in pregnant women were high-risk factors for NHB (Sup 1). Thus, we hypothesized if maternal serum TBA level and other indicators were associated with NHB-related neonatal amino acid metabolism disorders. We apply Spearman correlation analysis between maternal laboratory biochemical indices and amino acid levels in neonatal diagnosed with NHB. As shown in Sup 2, maternal TBA level was negatively associated with ALA (r=-0.167, *p* = 0.011), VAL (r=-0.214, *p* = 0.001), ORN (r=-0.196, *p* = 0.003), and PRO (r=-0.131, *p* = 0.045), meanwhile, higher ALT levels were associated with decreased levels of ALA (r=-0.135, *p* = 0.039), VAL (r=-0.177, *p* = 0.007), ORN (r=-0.257, *p* < 0.001), while ALT/AST was only positively correlated with ARG (r = 0.133, *p* = 0.013). Unfortunately, there was no significant correlation between maternal ALB, HDL, TC levels and neonatal amino acids (*p* > 0.05). In this regard, multivariate logistic regression analysis was used to further confirm whether serum TBA, ALT, and ALT/AST were significantly and independently correlated with NHB-related amino acid metabolism disorders (Table 3). As in previous reports, maternal gestational age, pre pregnancy BMI and gestational weight gain were risk factors for adverse pregnancy outcomes, and the significant differences were found in our study (Table 1), so these factors were adjusted for multivariate logistic regression analysis. After adjustment for confounding factors, neonatal ORN levels decreased with the independent increase of maternal TBA (adjusted odds ratio (AOR) = 0.379, 95%CI = 0.188–0.762, *p* = 0.006) and ALT levels (AOR = 0.441, 95%CI = 0.211–0.922, *p* = 0.030), respectively. Similarly, maternal ALT levels were an independent risk factor for neonatal VAL levels (AOR = 0.454, 95%CI = 0.218–0.949, *p* = 0.036). Based on the above results, our study indicated that maternal serum indicators TBA and ALT were independent risk factors for NHB-related amino acid metabolism disorders.

**Table 3:** Multiple regressions between neonatal amino acids and maternal laboratory biochemical indices

	ALA		VAL		ORN		ARG		PRO	
Variables	OR (95%CI)	*p*-value	OR (95%CI)	*p*-value	OR (95%CI)	*p*-value	OR (95%CI)	*p*-value	OR (95%CI)	*p*-value
TBA	0.707 (0.371–1.346)	0.291	0.556 (0.286–1.081)	0.084	0.379 (0.188–0.762)	0.006	1.011 (0.562–1.818)	0.971	0.650 (0.336–1.255)	0.199
ALT	0.840 (0.431–1.639)	0.610	0.454 (0.218–0.949)	0.036	0.441 (0.211–0.922)	0.030	1.052 (0.569–1.945)	0.871	1.488 (0.668–3.317)	0.331
ALT/AST	0.867 (0.438–1.715)	0.681	0.912 (0.459–1.814)	0.793	0.626 (0.303–1.293)	0.206	0.737 (0.399–1.364)	0.331	1.671 (0.863–3.234)	0.127

OR, odds ratio; AOR, adjusted odds ratio

Logistic regression analysis was adjusted for maternal gestational age, pre pregnancy BMI and gestational weight gain to determine the associations between neonatal amino acids and maternal laboratory biochemical indices

## Discussion

Elevated liver enzymes or excessive accumulation of bile acids during pregnancy can lead to adverse pregnancy outcomes in neonatal such as myocardial damage, hypoglycemia, and hyperbilirubinemia [11, 22, 23]. Previous reports have suggested that increased TBA and abnormal liver function in pregnant women may be risk factors for elevated bilirubin and TBA in neonatal, which can cause damage to the neonatal nervous system and severely impact the growth and development of the neonatal [24–26]. In our study, we investigated NHB and their pregnant mothers. Consistent with previous studies, we found that serum levels of TBA, ALT, AST, and ALT/AST in pregnant women in the NHB group were significantly higher than those in the control group, especially the TBA level was 6-fold higher than that in the control group, while ALB, TC, and HDL were lower. As demonstrated by Ceren Golbasi et al., a higher fT4 level was associated with a higher TBA level [27]. Thyroid hormones regulate multiple metabolic pathways in the body, especially lipoprotein metabolism. For example, an animal study confirmed that thyroid hormones play a biological function in the conversion of cholesterol to bile acids and bile secretion of cholesterol and bile acids [28, 29]. Moreover, a decreasing trend of LDL was found in the plasma of hyperthyroid patients [30]. Therefore, we hypothesized that the abnormal lipid metabolism of pregnant women in the NHB newborns may also be due to abnormal thyroid hormones.

As an important substance in human metabolism, amino acids play a significant role in the growth and development of neonatal [14]. The results of amino acids in NHB revealed a significant decrease in ALA, VAL, ORN, and PRO levels and an increase in ARG levels. ALA is synthesized by VAL and other branched chain amino acids after catabolism through the liver and released into the bloodstream, playing a role in repairing the damaged liver [15], whereas ORN and PRO can improve the liver function and enhance immunity [17, 18], respectively. The decrease of these amino acids in the NHB group suggested impaired liver function and self-healing ability. As a key amino acid to maintain positive nitrogen balance [19], the expression of ARG was elevated in our study, which might be a stress response of the body in disease conditions. Based on the positive correlation between thyroid hormones and TBA concentrations found by Ceren Golbasi et al. [27], we also hypothesized that with the accumulation of TBA, abnormal thyroid hormone may also lead to the disorder of amino acid metabolism because of the thyroid hormone’s function of promoting amino acid catabolism [31, 32]. However, due to the lack of data on thyroid hormones, we were unable to test this hypothesis to further elucidate the mechanism. Undeniably, more clinical data, such as the levels of endocrine-related hormones in mothers and their newborn baby, neonatal blood ammonia and other data were needed to verify this theory. In conclusion, our study revealed amino acid disturbances in NHB, as well as abnormalities in liver enzymes, TBA, and lipid metabolism in pregnant women.

In clinical, heel blood is usually collected from newborns 3–4 days after birth to detect amino acid levels. It will be valuable for early monitoring and intervention for NHB by predicting amino acid metabolism levels of newborns through maternal serum indicators during pregnancy. Therefore, we used correlation analysis and logistic regression to analyze the levels of maternal serum indicators and the risk of NHB and NHB-related amino acid metabolism disorders. The data showed that maternal levels of ALT, AST, ALT/AST, and TBA were all the high-risk factors of NHB, however, a negative relationship was found between ALB, TC, HDL, and NHB respectively. Correlation analysis showed a decrease in both ALA, VAL, ORN and PRO levels as maternal TBA levels increased, and the same trend we found in ALT with ALA, VAL ORN, while ALT/AST was positively correlated with ARG only. After further logistic regression adjustment for confounders such as maternal gestational age, pre-pregnancy BMI, and gestational weight gain, we still found that high levels of maternal TBA and ALT were risk factors for ORN, while high levels of ALT were also risk factors for VAL.

In conclusion, we demonstrate that the levels of maternal serum TBA, ALT, and AST were significantly higher, while lipid metabolism indicators of HDL and TC were lower in the NHB group than those in the No-NHB group, which may be associated with the development of NHB. In addition, our results showed that the levels of maternal TBA and ALT are significantly and positively associated with reduced levels of NHB-related amino acids. Of course, our research has certain shortcomings, such as the lack of data on the impact of medication on outcomes in pregnant women, as well as the influence of newborn breastfeeding mode and colostrum time on postnatal amino acid metabolism levels [33], which may bring certain biases to our study results. However, our study to some extent indicates significant differences in blood amino acid levels between NHB and non-NHB, and the detection of maternal serum TBA and ALT is expected to be a method for monitoring NHB-related amino acid metabolic disorders, contributing to the early detection of fetal anomalies, the reduction of complications and other adverse pregnancy outcomes, and improvement of prognosis for the affected neonatal.

### Electronic supplementary material

Below is the link to the electronic supplementary material.

Supplementary Material 1

## Data Availability

The data that support the findings of this study are available on request from the corresponding author. The data are not publicly available due to privacy or ethical restrictions.
