# Supplementary material for: The correlation between serum total bile acid and alanine aminotransferase of pregnant women and the disorders of neonatal hyperbilirubinemia-related amino acid metabolism
Source: BMC Pregnancy Childbirth. 2024 Jan 3;24:26. doi: 10.1186/s12884-023-06226-9 (PMC10763467; doi:10.1186/s12884-023-06226-9)
Supplement: Supplementary file 1 — Supplementary Material 1 [file 12884_2023_6226_MOESM1_ESM.doc]

**Supplementary Material**


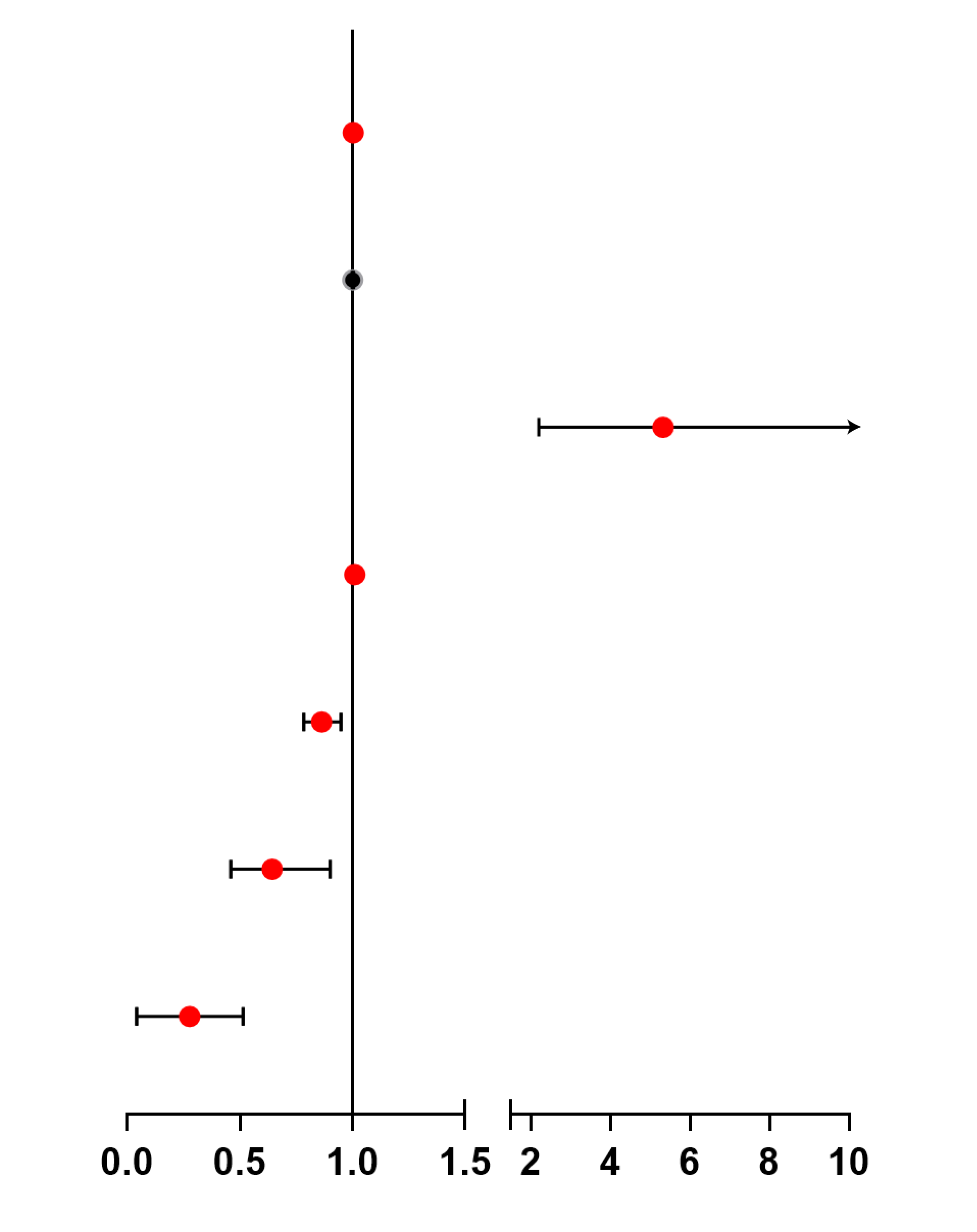
**Supplementary Material 1: Associations between** **neonatal hyperbilirubinemia and maternal laboratory biochemical indices**

|  | Model 1 | | | Model 2 | |
| --- | --- | --- | --- | --- | --- |
| Variables | OR (95%CI) | *p*-value | AOR (95%CI) | | *p*-value |
| ALT (U/L) | 1.003 (1.001-1.006) | 0.019 | 1.003 (1.000-1.006) | | 0.024 |
| AST(U/L) | 1.002 (0.999-1.005) | 0.110 | 1.002 (1.000-1.005) | | 0.102 |
| ALT/AST | 5.483 (2.373-12.670) | 0.000 | 5.321 (2.201-12.864) | | 0.000 |
| TBA (μmol/L) | 1.011 (1.002-1.020) | 0.017 | 1.010 (1.001-1.019) | | 0.031 |
| ALB (g/L) | 0.854 (0.779-0.937) | 0.001 | 0.862 (0.784-0.948) | | 0.002 |
| TC (mmol/L) | 0.673 (0.486-0.933) | 0.017 | 0.644 (0.460-0.902) | | 0.010 |
| HDL (mmol/L) | 0.211 (0.084-0.532) | 0.001 | 0.210 (0.082-0.541) | | 0.001 |

OR, odds ratio; AOR, adjusted odds ratio.

Logistic regression analysis was performed to determine the associations between neonatal hyperbilirubinemia and maternal laboratory biochemical indices. Model 1 was unadjusted for confounding factors; Model 2 was adjusted for maternal gestational age, pre pregnancy BMI and gestational weight gain. The forest map was a visualization of Model 2 and the red dots represent *p*< 0.05.

**Supplementary Material 2:** Correlation analysis between neonatal amino acids and maternal laboratory biochemical indices

| Variables | TBA | | ALT | | ALT/AST | | ALB | | HDL | | TC | |
| --- | --- | --- | --- | --- | --- | --- | --- | --- | --- | --- | --- | --- |
|  | *r* | *p* | *r* | *p* | *r* | *p* | *r* | *p* | *r* | *p* | *r* | *p* |
|  |  |  |  |  |  |  |  |  |  |  |  |  |
| ALA | -0.167 | 0.011 | -0.135 | 0.039 | -0.082 | 0.214 | 0.078 | 0.247 | -0.090 | 0.235 | -0.081 | 0.288 |
| VAL | -0.214 | 0.001 | -0.177 | 0.007 | -0.058 | 0.375 | 0.099 | 0.144 | -0.074 | 0.331 | -0.104 | 0.168 |
| ORN | -0.196 | 0.003 | -0.254 | 0.000 | -0.097 | 0.141 | 0.104 | 0.124 | 0.112 | 0.140 | 0.010 | 0.891 |
| ARG | 0.064 | 0.333 | 0.064 | 0.334 | 0.133 | 0.013 | -0.021 | 0.756 | -0.018 | 0.808 | -0.070 | 0.358 |
| PRO | -0.131 | 0.045 | -0.066 | 0.317 | 0.001 | 0.993 | 0.123 | 0.070 | 0.056 | 0.462 | -0.052 | 0.497 |

The Spearman correlation analysis were performed to assess the correlation between neonatal amino acids and maternal laboratory biochemical indices.
